# *catena*-Poly[[(2-{[2-(dimethyl­ammonio)­eth­yl]imino­meth­yl}pyridine-κ^2^ *N*,*N*′)bis­(thio­cyanato-κ*N*)manganese(II)]-μ-thio­cyanato-κ^2^ *N*:*S*]

**DOI:** 10.1107/S1600536812032874

**Published:** 2012-07-28

**Authors:** Jun Wang, Wubiao Zhu, Jichang Li

**Affiliations:** a Zhongshan Polytechnic, Zhongshan, Guangdong 528404, People’s Republic of China

## Abstract

In the title one-dimensional coordination polymer, [Mn(NCS)_3_(C_10_H_16_N_3_)]_*n*_, the Mn^II^ atom is coordinated by an *N*,*N*′-bidentate Schiff base and four thio­cyanate ligands in a distorted octa­hedral N_5_S geometry. Bridging thio­cyanate ligands inter­connect adjacent [Mn(NCS)_2_(C_10_H_16_N_3_)] units, giving rise to helical chains extending along the *b* axis. The chains are further linked through N—H⋯S hydrogen bonds, leading to a three-dimensional supra­molecular network.

## Related literature

For the structure of Cu^II^ and Pt^I^ complexes of the same Schiff base, see: Hinman *et al.* (2000 ▶); Mukherjee *et al.* (2002 ▶).
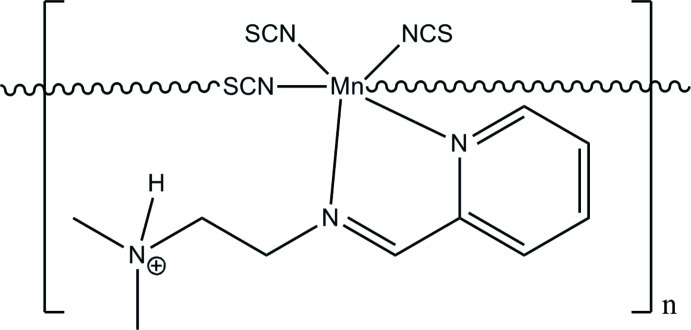


## Experimental

### 

#### Crystal data

[Mn(NCS)_3_(C_10_H_16_N_3_)]
*M* *_r_* = 407.44
Orthorhombic, 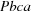

*a* = 8.5603 (12) Å
*b* = 11.0699 (15) Å
*c* = 37.346 (5) Å
*V* = 3539.0 (8) Å^3^
*Z* = 8
Mo *K*α radiation
μ = 1.11 mm^−1^
*T* = 296 K
0.25 × 0.19 × 0.11 mm

#### Data collection

Bruker APEXII area-detector diffractometer
Absorption correction: multi-scan (*SADABS*; Sheldrick, 1996 ▶) *T*_min_ = 0.770, *T*_max_ = 0.888
18847 measured reflections
3660 independent reflections
2397 reflections with *I* > 2σ(*I*)
*R*_int_ = 0.076

#### Refinement

*R*[*F* ^2^ > 2σ(*F* ^2^)] = 0.043
*wR*(*F* ^2^) = 0.098
*S* = 1.04
3660 reflections
210 parameters
H-atom parameters constrained
Δρ_max_ = 0.29 e Å^−3^
Δρ_min_ = −0.35 e Å^−3^

### 

Data collection: *APEX2* (Bruker, 2004 ▶); cell refinement: *SAINT* (Bruker, 2004 ▶); data reduction: *SAINT*; program(s) used to solve structure: *SHELXS97* (Sheldrick, 2008 ▶); program(s) used to refine structure: *SHELXL97* (Sheldrick, 2008 ▶); molecular graphics: *SHELXTL* (Sheldrick, 2008 ▶); software used to prepare material for publication: *SHELXTL*.

## Supplementary Material

Crystal structure: contains datablock(s) I, global. DOI: 10.1107/S1600536812032874/rz2792sup1.cif

Structure factors: contains datablock(s) I. DOI: 10.1107/S1600536812032874/rz2792Isup2.hkl

Supplementary material file. DOI: 10.1107/S1600536812032874/rz2792Isup3.mol

Additional supplementary materials:  crystallographic information; 3D view; checkCIF report

## Figures and Tables

**Table 1:** Hydrogen-bond geometry (Å, °)

*D*—H⋯*A*	*D*—H	H⋯*A*	*D*⋯*A*	*D*—H⋯*A*
N3—H3⋯S1^i^	0.87	2.47	3.294 (3)	159

Symmetry code: (i) 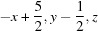.

**Fig. 1.**
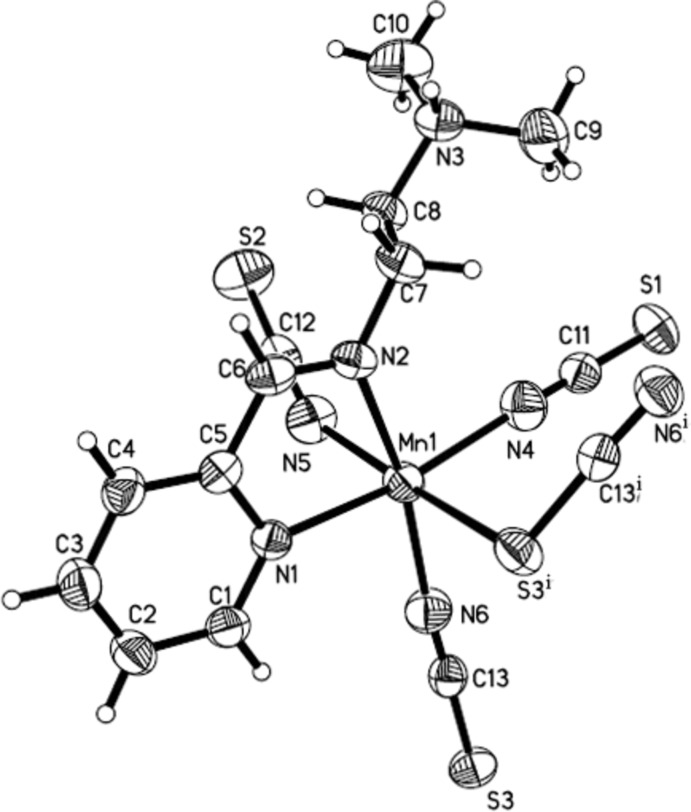
The asymmetric unit of the title compound with displacement ellipsoids drawn at the 50% probability level. Symmetry code: (i) 2.5-x, 0.5+y, z.

## supplementary crystallographic information

### Comment

The title compound (Fig. 1) was obtained upon complexation of the Schiff base *N*,*N*-dimethyl-*N*'-[(pyridin-2-yl)methylene]ethane-1,2-diamine with Mn(ClO_4_)_2_ and KNCS. Similarly to what observed in a related platinium(II) complex (Hinman *et al.*, 2000), due to the protonation of the amine nitrogen atom the Schiff base acts as a bidentate ligand instead as tridentate (Mukherjee *et al.*, 2002). The Mn(II) ion is in a distorted octahedral coordination environment, provided by an *N*,*N*'-bidentate Schiff base and four NCS ligands. The µ_2_-isothiocyanato ligands interconnect the [Mn(NCS)_2_(C_10_H_16_N_3_)] units, giving rise to one-dimensional helical chains along the *b* axis. Adjacent helical chains are further connected *via* N—H···S hydrogen bonds (Table 1) into a three-dimensional supramolecular structure.

### Experimental

A mixture of 2-pyridinecarboxaldehyde (0.107 g, 1 mmol) and *N*,*N*-dimethylethyldiamine (0.088 g, 1 mmol) in ethanol (5 ml) was refluxed for 2 h followed by addition of a solution of Mn(ClO_4_)_2_.6H_2_O (0.362 g, 1 mmol) and KNCS (0.291, 3 mmol) in a minimum amount of water. The resulting solution was refluxed for 30 min, then set aside at room temperature. Crystals of the title compound suitable for X-ray analysis were obtained after few days on slow evaporation of the solvent.

### Refinement

Hydrogen atoms were located in a difference Fourier map or placed at calculated positions (C—H = 0.95–0.99 Å; N—H = 0.87 Å), and were treated as riding on their parent atoms, with *U*_iso_(H) = 1.2 *U*_eq_(C) or 1.5 *U*_eq_(C, N) for amine and methyl H atoms.

### Crystal data

**Table:**

[Mn(NCS)_3_(C_10_H_16_N_3_)]	*F*(000) = 1672
*M**_r_* = 407.44	*D*_x_ = 1.529 Mg m^−^^3^
Orthorhombic, *P**b**c**a*	Mo *K*α radiation, λ = 0.71073 Å
Hall symbol: -P 2ac 2ab	Cell parameters from 3600 reflections
*a* = 8.5603 (12) Å	θ = 1.2–28.0°
*b* = 11.0699 (15) Å	µ = 1.11 mm^−^^1^
*c* = 37.346 (5) Å	*T* = 296 K
*V* = 3539.0 (8) Å^3^	Block, yellow
*Z* = 8	0.25 × 0.19 × 0.11 mm

### Data collection

**Table:**

Bruker APEXII area-detector diffractometer	3660 independent reflections
Radiation source: fine-focus sealed tube	2397 reflections with *I* > 2σ(*I*)
Graphite monochromator	*R*_int_ = 0.076
φ and ω scan	θ_max_ = 26.5°, θ_min_ = 2.2°
Absorption correction: multi-scan (*SADABS*; Sheldrick, 1996)	*h* = −10→10
*T*_min_ = 0.770, *T*_max_ = 0.888	*k* = −13→12
18847 measured reflections	*l* = −46→44

### Refinement

**Table:**

Refinement on *F*^2^	Primary atom site location: structure-invariant direct methods
Least-squares matrix: full	Secondary atom site location: difference Fourier map
*R*[*F*^2^ > 2σ(*F*^2^)] = 0.043	Hydrogen site location: inferred from neighbouring sites
*wR*(*F*^2^) = 0.098	H-atom parameters constrained
*S* = 1.04	*w* = 1/[σ^2^(*F*_o_^2^) + (0.0371*P*)^2^ + 0.1816*P*] where *P* = (*F*_o_^2^ + 2*F*_c_^2^)/3
3660 reflections	(Δ/σ)_max_ = 0.001
210 parameters	Δρ_max_ = 0.29 e Å^−^^3^
0 restraints	Δρ_min_ = −0.35 e Å^−^^3^

### Special details

**Table:**

Geometry. All e.s.d.'s (except the e.s.d. in the dihedral angle between two l.s. planes) are estimated using the full covariance matrix. The cell e.s.d.'s are taken into account individually in the estimation of e.s.d.'s in distances, angles and torsion angles; correlations between e.s.d.'s in cell parameters are only used when they are defined by crystal symmetry. An approximate (isotropic) treatment of cell e.s.d.'s is used for estimating e.s.d.'s involving l.s. planes.
Refinement. Refinement of *F*^2^ against ALL reflections. The weighted *R*-factor *wR* and goodness of fit *S* are based on *F*^2^, conventional *R*-factors *R* are based on *F*, with *F* set to zero for negative *F*^2^. The threshold expression of *F*^2^ > σ(*F*^2^) is used only for calculating *R*-factors(gt) *etc*. and is not relevant to the choice of reflections for refinement. *R*-factors based on *F*^2^ are statistically about twice as large as those based on *F*, and *R*- factors based on ALL data will be even larger.

### Fractional atomic coordinates and isotropic or equivalent isotropic displacement parameters (Å^2^)

**Table:**

	*x*	*y*	*z*	*U*_iso_*/*U*_eq_	
C1	0.8026 (4)	1.0515 (3)	0.29186 (7)	0.0365 (7)	
H1	0.8775	1.1075	0.2848	0.044*	
C2	0.6523 (4)	1.0652 (3)	0.27924 (8)	0.0427 (8)	
H2	0.6263	1.1293	0.2643	0.051*	
C3	0.5419 (4)	0.9820 (3)	0.28924 (8)	0.0470 (9)	
H3A	0.4396	0.9885	0.2811	0.056*	
C4	0.5847 (4)	0.8885 (3)	0.31150 (8)	0.0390 (8)	
H4	0.5115	0.8311	0.3185	0.047*	
C5	0.7368 (4)	0.8806 (3)	0.32342 (7)	0.0328 (7)	
C6	0.7882 (4)	0.7845 (3)	0.34732 (8)	0.0365 (8)	
H6	0.7188	0.7237	0.3539	0.044*	
C7	0.9714 (4)	0.6847 (3)	0.38312 (8)	0.0400 (8)	
H7A	1.0726	0.6528	0.3762	0.048*	
H7B	0.8954	0.6198	0.3818	0.048*	
C8	0.9784 (4)	0.7345 (3)	0.42070 (8)	0.0381 (8)	
H8A	1.0409	0.8076	0.4207	0.046*	
H8B	0.8736	0.7561	0.4283	0.046*	
C9	1.2174 (4)	0.6312 (3)	0.44276 (10)	0.0601 (11)	
H9A	1.2693	0.7068	0.4469	0.090*	
H9B	1.2399	0.6036	0.4189	0.090*	
H9C	1.2538	0.5726	0.4598	0.090*	
C10	1.0076 (5)	0.6853 (4)	0.48428 (8)	0.0679 (12)	
H10A	1.0542	0.6297	0.5009	0.102*	
H10B	0.8963	0.6854	0.4874	0.102*	
H10C	1.0477	0.7650	0.4885	0.102*	
C11	1.3790 (4)	0.8798 (3)	0.39279 (8)	0.0358 (7)	
C12	0.9167 (4)	1.0407 (3)	0.41865 (10)	0.0411 (8)	
C13	1.2401 (4)	1.1848 (3)	0.30617 (8)	0.0351 (7)	
Mn1	1.07355 (5)	0.94723 (4)	0.344285 (12)	0.03482 (15)	
N1	0.8468 (3)	0.9621 (2)	0.31373 (6)	0.0312 (6)	
N2	0.9266 (3)	0.7830 (2)	0.35923 (6)	0.0335 (6)	
N3	1.0459 (3)	0.6475 (2)	0.44693 (7)	0.0398 (7)	
N4	1.2709 (3)	0.8875 (2)	0.37436 (7)	0.0469 (7)	
N5	0.9625 (4)	1.0375 (2)	0.38952 (8)	0.0500 (8)	
N6	1.1752 (3)	1.1042 (2)	0.31952 (7)	0.0447 (7)	
H3	0.9996	0.5806	0.4412	0.067*	
S1	1.53139 (11)	0.87179 (8)	0.41894 (2)	0.0482 (3)	
S2	0.85784 (14)	1.04061 (10)	0.46007 (2)	0.0646 (3)	
S3	1.32720 (10)	1.29615 (7)	0.28601 (2)	0.0427 (2)	

### Atomic displacement parameters (Å^2^)

**Table:**

	*U*^11^	*U*^22^	*U*^33^	*U*^12^	*U*^13^	*U*^23^
C1	0.040 (2)	0.0343 (18)	0.0352 (17)	0.0006 (15)	0.0050 (15)	0.0024 (15)
C2	0.045 (2)	0.042 (2)	0.0411 (19)	0.0111 (17)	−0.0011 (17)	0.0056 (16)
C3	0.037 (2)	0.060 (2)	0.044 (2)	0.0059 (18)	−0.0041 (17)	0.0013 (18)
C4	0.0304 (19)	0.044 (2)	0.0424 (19)	−0.0054 (15)	0.0004 (15)	0.0008 (16)
C5	0.0332 (19)	0.0328 (18)	0.0325 (17)	−0.0029 (14)	0.0030 (14)	−0.0017 (14)
C6	0.044 (2)	0.0306 (18)	0.0350 (17)	−0.0069 (14)	0.0037 (16)	0.0018 (14)
C7	0.050 (2)	0.0276 (17)	0.0426 (19)	0.0029 (15)	−0.0033 (16)	0.0048 (15)
C8	0.047 (2)	0.0307 (18)	0.0366 (18)	0.0059 (15)	−0.0006 (16)	0.0037 (14)
C9	0.048 (2)	0.067 (3)	0.065 (3)	0.003 (2)	−0.009 (2)	0.005 (2)
C10	0.094 (3)	0.078 (3)	0.032 (2)	−0.003 (2)	0.002 (2)	−0.006 (2)
C11	0.0377 (19)	0.0300 (18)	0.0398 (19)	−0.0024 (14)	0.0013 (16)	−0.0002 (15)
C12	0.038 (2)	0.0301 (18)	0.055 (2)	−0.0001 (15)	−0.0126 (18)	−0.0077 (17)
C13	0.0318 (19)	0.0372 (19)	0.0362 (17)	−0.0002 (14)	−0.0020 (15)	−0.0036 (15)
Mn1	0.0324 (3)	0.0317 (3)	0.0404 (3)	−0.0035 (2)	−0.0037 (2)	0.0040 (2)
N1	0.0315 (15)	0.0291 (14)	0.0328 (14)	0.0003 (11)	0.0012 (11)	0.0017 (12)
N2	0.0421 (17)	0.0280 (14)	0.0304 (14)	0.0007 (12)	−0.0001 (12)	0.0023 (11)
N3	0.0482 (19)	0.0363 (15)	0.0349 (14)	−0.0042 (13)	−0.0040 (13)	0.0010 (12)
N4	0.0424 (18)	0.0521 (18)	0.0463 (17)	−0.0019 (14)	−0.0078 (15)	−0.0031 (14)
N5	0.058 (2)	0.0441 (18)	0.0483 (18)	−0.0010 (14)	0.0039 (16)	−0.0054 (15)
N6	0.0447 (18)	0.0370 (16)	0.0525 (18)	−0.0050 (14)	0.0026 (14)	0.0029 (14)
S1	0.0476 (6)	0.0432 (5)	0.0538 (5)	0.0057 (4)	−0.0176 (4)	−0.0027 (4)
S2	0.0818 (8)	0.0664 (7)	0.0455 (6)	−0.0123 (6)	0.0038 (5)	−0.0092 (5)
S3	0.0478 (6)	0.0360 (5)	0.0442 (5)	−0.0086 (4)	0.0037 (4)	0.0034 (4)

### Geometric parameters (Å, º)

**Table:**

C1—N1	1.337 (3)	C9—H9A	0.9600
C1—C2	1.379 (4)	C9—H9B	0.9600
C1—H1	0.9300	C9—H9C	0.9600
C2—C3	1.371 (5)	C10—N3	1.492 (4)
C2—H2	0.9300	C10—H10A	0.9600
C3—C4	1.377 (4)	C10—H10B	0.9600
C3—H3A	0.9300	C10—H10C	0.9600
C4—C5	1.379 (4)	C11—N4	1.157 (4)
C4—H4	0.9300	C11—S1	1.632 (3)
C5—N1	1.354 (4)	C12—N5	1.157 (4)
C5—C6	1.457 (4)	C12—S2	1.627 (4)
C6—N2	1.266 (4)	C13—N6	1.162 (4)
C6—H6	0.9300	C13—S3	1.626 (3)
C7—N2	1.459 (3)	Mn1—N4	2.133 (3)
C7—C8	1.509 (4)	Mn1—N6	2.153 (3)
C7—H7A	0.9700	Mn1—N5	2.181 (3)
C7—H7B	0.9700	Mn1—N1	2.257 (2)
C8—N3	1.490 (4)	Mn1—N2	2.280 (2)
C8—H8A	0.9700	Mn1—S3^i^	2.8731 (10)
C8—H8B	0.9700	N3—H3	0.8675
C9—N3	1.487 (4)	S3—Mn1^ii^	2.8732 (10)
			
N1—C1—C2	123.6 (3)	H10A—C10—H10B	109.5
N1—C1—H1	118.2	N3—C10—H10C	109.5
C2—C1—H1	118.2	H10A—C10—H10C	109.5
C3—C2—C1	118.4 (3)	H10B—C10—H10C	109.5
C3—C2—H2	120.8	N4—C11—S1	178.9 (3)
C1—C2—H2	120.8	N5—C12—S2	177.5 (3)
C2—C3—C4	119.0 (3)	N6—C13—S3	177.7 (3)
C2—C3—H3A	120.5	N4—Mn1—N6	98.99 (11)
C4—C3—H3A	120.5	N4—Mn1—N5	94.55 (11)
C3—C4—C5	119.6 (3)	N6—Mn1—N5	98.00 (10)
C3—C4—H4	120.2	N4—Mn1—N1	165.80 (10)
C5—C4—H4	120.2	N6—Mn1—N1	94.10 (9)
N1—C5—C4	121.9 (3)	N5—Mn1—N1	89.04 (10)
N1—C5—C6	116.1 (3)	N4—Mn1—N2	93.50 (10)
C4—C5—C6	122.0 (3)	N6—Mn1—N2	166.41 (10)
N2—C6—C5	120.5 (3)	N5—Mn1—N2	86.27 (10)
N2—C6—H6	119.8	N1—Mn1—N2	72.99 (9)
C5—C6—H6	119.8	N4—Mn1—S3^i^	89.10 (8)
N2—C7—C8	107.8 (2)	N6—Mn1—S3^i^	91.43 (7)
N2—C7—H7A	110.1	N5—Mn1—S3^i^	169.22 (8)
C8—C7—H7A	110.1	N1—Mn1—S3^i^	85.06 (6)
N2—C7—H7B	110.1	N2—Mn1—S3^i^	83.38 (6)
C8—C7—H7B	110.1	C1—N1—C5	117.3 (3)
H7A—C7—H7B	108.5	C1—N1—Mn1	127.3 (2)
N3—C8—C7	113.0 (2)	C5—N1—Mn1	114.49 (19)
N3—C8—H8A	109.0	C6—N2—C7	118.1 (3)
C7—C8—H8A	109.0	C6—N2—Mn1	114.85 (19)
N3—C8—H8B	109.0	C7—N2—Mn1	126.8 (2)
C7—C8—H8B	109.0	C9—N3—C10	110.4 (3)
H8A—C8—H8B	107.8	C9—N3—C8	113.1 (3)
N3—C9—H9A	109.5	C10—N3—C8	110.4 (3)
N3—C9—H9B	109.5	C9—N3—H3	108.7
H9A—C9—H9B	109.5	C10—N3—H3	111.8
N3—C9—H9C	109.5	C8—N3—H3	102.2
H9A—C9—H9C	109.5	C11—N4—Mn1	165.9 (3)
H9B—C9—H9C	109.5	C12—N5—Mn1	152.9 (3)
N3—C10—H10A	109.5	C13—N6—Mn1	175.2 (3)
N3—C10—H10B	109.5	C13—S3—Mn1^ii^	103.08 (11)
			
N1—C1—C2—C3	−0.8 (5)	C5—C6—N2—Mn1	5.0 (4)
C1—C2—C3—C4	0.3 (5)	C8—C7—N2—C6	−104.8 (3)
C2—C3—C4—C5	0.3 (5)	C8—C7—N2—Mn1	69.3 (3)
C3—C4—C5—N1	−0.3 (5)	N4—Mn1—N2—C6	177.1 (2)
C3—C4—C5—C6	179.3 (3)	N6—Mn1—N2—C6	−26.1 (5)
N1—C5—C6—N2	3.2 (4)	N5—Mn1—N2—C6	82.8 (2)
C4—C5—C6—N2	−176.4 (3)	N1—Mn1—N2—C6	−7.4 (2)
N2—C7—C8—N3	−171.4 (3)	S3^i^—Mn1—N2—C6	−94.2 (2)
C2—C1—N1—C5	0.8 (4)	N4—Mn1—N2—C7	2.8 (2)
C2—C1—N1—Mn1	−167.9 (2)	N6—Mn1—N2—C7	159.5 (4)
C4—C5—N1—C1	−0.2 (4)	N5—Mn1—N2—C7	−91.6 (2)
C6—C5—N1—C1	−179.9 (2)	N1—Mn1—N2—C7	178.3 (2)
C4—C5—N1—Mn1	169.9 (2)	S3^i^—Mn1—N2—C7	91.4 (2)
C6—C5—N1—Mn1	−9.8 (3)	C7—C8—N3—C9	72.4 (4)
N4—Mn1—N1—C1	−163.6 (4)	C7—C8—N3—C10	−163.3 (3)
N6—Mn1—N1—C1	−6.4 (2)	N6—Mn1—N4—C11	48.5 (11)
N5—Mn1—N1—C1	91.5 (2)	N5—Mn1—N4—C11	−50.4 (11)
N2—Mn1—N1—C1	177.9 (2)	N1—Mn1—N4—C11	−154.6 (9)
S3^i^—Mn1—N1—C1	−97.5 (2)	N2—Mn1—N4—C11	−136.9 (11)
N4—Mn1—N1—C5	27.5 (5)	S3^i^—Mn1—N4—C11	139.8 (11)
N6—Mn1—N1—C5	−175.4 (2)	N4—Mn1—N5—C12	−50.7 (6)
N5—Mn1—N1—C5	−77.4 (2)	N6—Mn1—N5—C12	−150.4 (6)
N2—Mn1—N1—C5	8.97 (19)	N1—Mn1—N5—C12	115.6 (6)
S3^i^—Mn1—N1—C5	93.54 (19)	N2—Mn1—N5—C12	42.5 (6)
C5—C6—N2—C7	179.9 (2)	S3^i^—Mn1—N5—C12	58.8 (9)

Symmetry codes: (i) −*x*+5/2, *y*−1/2, *z*; (ii) −*x*+5/2, *y*+1/2, *z*.

### Hydrogen-bond geometry (Å, º)

**Table:**

*D*—H···*A*	*D*—H	H···*A*	*D*···*A*	*D*—H···*A*
N3—H3···S1^i^	0.87	2.47	3.294 (3)	159

Symmetry code: (i) −*x*+5/2, *y*−1/2, *z*.
